# Editorial: Anthocyanins in nutritional science

**DOI:** 10.3389/fnut.2026.1844300

**Published:** 2026-05-20

**Authors:** Lahcen Hssaini, Rachida Ouaabou, Mirela Kopjar

**Affiliations:** 1 Agro-Food Technology and Quality Laboratory, Regional Center of Agricultural Research of Meknes, National Institute of Agricultural Research, Rabat, Morocco; 2 Équipe de recherche en chimie, informatique et intelligence artificielle(ERCI2A), FSTH, Abdelmalek Essaadi University, Tetouan, Morocco; 3 Faculty of Food Technology, Franjo Kuhač, Osijek, Croatia

**Keywords:** anthocyanins, anthocyanins-based fortification, antioxidant activity, bioavailability, cardiometabolic health, chronic disease prevention, dietary source, phytochemicals

## Introduction

Anthocyanins, the water-soluble flavonoid pigments responsible for the vibrant red, purple, and blue hues in the plant kingdom, have transcended their role as mere natural colorants to become a focal point of modern nutritional science. Historically, these compounds served as evolutionary adaptations, protecting plants from environmental stressors such as ultraviolet radiation and extreme temperatures. Today, a growing body of evidence suggests that these same protective properties confer profound health benefits to humans, particularly in the context of aging and chronic disease prevention.

Despite the well-documented potential of anthocyanins, ranging from antioxidant capacity to anti-inflammatory modulation, significant challenges remain in their “soil-to-cell” journey. The efficacy of these compounds is often limited by agricultural variability, instability during food processing, and complex bioavailability pathways in the human body. As the global burden of age-related pathologies such as neurodegenerative disorders, cardiovascular disease, and metabolic syndrome continues to rise, the need for a multidisciplinary approach to anthocyanin research has never been more urgent.

This Research Topic, “*Anthocyanins in Nutritional Science*” was curated to bridge the critical gaps between agricultural biofortification, food engineering, and clinical application. By integrating original research on cultivation strategies, encapsulation technologies, and industrial stability with comprehensive reviews of therapeutic mechanisms, this Research Topic offers a holistic perspective on how to optimize the production, stability, and delivery of these vital phytonutrients.

## Therapeutic horizons: combating the hallmarks of aging

The cornerstone of this Research Topic is the comprehensive review by Ma et al., which systematically categorizes the health benefits of anthocyanins against age-related diseases. Aging is intrinsically linked to oxidative stress, chronic inflammation, and cellular senescence—processes that drive the deterioration of physiological function. Ma et al. highlight that anthocyanins intervene in these processes through pleiotropic mechanisms, including the activation of autophagy and the regulation of the gut microbiota.

In the realm of neurodegenerative diseases, the synthesis provided by Ma et al. shows that anthocyanins can alleviate cognitive deficits associated with Alzheimer's and Parkinson's diseases. Mechanistically, these compounds have been shown to regulate the PI3K/Akt/GSK3β pathway, thereby reducing reactive oxygen species (ROS) and inhibiting neuronal apoptosis. Furthermore, the authors discuss the critical role of anthocyanins in cardiovascular health, noting their ability to improve endothelial function and reduce arterial stiffness. Crucially, their work also explores the potential of anthocyanins to mitigate age-related bone loss and metabolic syndrome, presenting a multi-target therapeutic strategy for healthspan extension.

## Biofortification: the synergy of minerals and pigments

To realize these therapeutic benefits, we must first ensure the abundance and quality of anthocyanins in our food supply. Wang et al. address this challenge through an agronomic lens, exploring the biofortification of purple leaf mustard (*Brassica juncea*). Their research tackles the “growth-defense” dilemma, where increasing secondary metabolites often comes at the cost of plant biomass.

In their contribution, Wang et al. demonstrate that preharvest treatment with sodium selenite is a viable strategy to enhance nutritional quality without compromising yield. Their study reveals that a specific concentration of 10 mg/kg of selenium acts as a physiological “sweet spot,” promoting root system development while significantly upregulating the expression of 12 key genes in the phenylpropanoid pathway, such as *PAL, CHS*, and *F3H*. This transcriptomic response led to a significant increase in total anthocyanin content (TAC) and improved the plant's overall antioxidant enzyme activities (APX, CAT, and POD). This research provides a scalable model for precision agriculture, suggesting that selenium biofortification can simultaneously boost the bioactive profile and mineral density of functional crops.

## Delivery and industrial stability: from microencapsulation to viticulture

Even when successfully grown, the instability of anthocyanins during processing and storage remains a significant hurdle. These pigments are notoriously sensitive to pH, temperature, and light. Two articles in this Research Topic address this from different industrial perspectives.

Muñoz-Coyotecatl et al. present a food engineering solution using complex coacervation to microencapsulate anthocyanins from red cabbage. By utilizing a ternary blend of pea protein, gum arabic, and the prebiotic inulin, they achieved encapsulation efficiency levels exceeding 95%. Their study is particularly notable for integrating inulin, which not only provides a structural matrix but also offers functional value for gut health—a synergy that aligns with the therapeutic mechanisms discussed by Ma et al. Their microcapsules retained biological functionality, demonstrating a 22.48% inhibition of α-amylase activity, which suggests potential applications in the management of postprandial glycemic responses through a clean-label delivery system.

Complementing this technological approach, Yue et al. investigated the stability of anthocyanins within a complex food matrix: red wine. In their study, they explored how the addition of mannoprotein influences the color stability and antioxidant capacity of Cabernet Sauvignon during the aging process. Their findings reveal that mannoprotein treatment effectively preserved the total phenolic and anthocyanin contents while stabilizing the wine's characteristic redness and brightness (L^*^, a^*^, b^*^ CIELAB color space parameters). By preventing the degradation and precipitation of these pigments through interactions with phenolic acids, Yue et al. demonstrate how specific food-grade additives can ensure that the antioxidant potential of anthocyanins remains intact from the point of production to the point of consumption.

## Thematic synthesis: a holistic value chain

When viewed collectively, the articles in this Research Topic establish a comprehensive value chain for anthocyanins in nutritional science. The work begins at the genomic and agronomic level with Wang et al., who demonstrate how targeted mineral interventions can prime plants for higher nutrient density. Once harvested, the challenges of stability are met by the innovative encapsulation techniques of Muñoz-Coyotecatl et al. and the matrix-stabilization strategies of Yue et al. These technical advancements ensure that the bioactive compounds survive the rigors of the food industry to reach the consumer in a functional state. Finally, the clinical framework provided by Ma et al. gives meaning to these efforts, detailing the specific biological pathways through which these stabilized pigments can combat the debilitating effects of aging and chronic disease.

## Conclusion and future directions

The research compiled in this Research Topic illustrates that the journey of anthocyanins, from the field to the industrial product and finally to the human body, is a complex interplay of agriculture, chemistry, and physiology.

As we stand at a pivotal moment in nutritional science, the evidence is clear: anthocyanins are not merely aesthetic components of our diet but are essential tools for healthspan extension. Future research must now focus on bridging these domains further—translating agronomic successes into large-scale production, refining delivery systems for maximum bioavailability in the upper gastrointestinal tract, and conducting longitudinal clinical trials to cement the role of anthocyanins in medical nutrition therapy.

